# Evaluation of an automated far ultraviolet-C light technology for decontamination of surfaces and aerosolized viruses in bathrooms

**DOI:** 10.1186/s13756-024-01473-7

**Published:** 2024-09-29

**Authors:** Claire E. Kaple, Samir Memic, Jennifer L. Cadnum, Curtis J. Donskey

**Affiliations:** 1 Department of Molecular Biology and Microbiology, Case Western Reserve University School of Medicine, Cleveland, OH USA; 2 Research Service, Louis Stokes Cleveland VA Medical Center, Cleveland, OH USA; 3 Department of Systems Biology, Case Western Reserve University School of Medicine, Cleveland, OH USA; 4 Department of Medicine, Case Western Reserve University School of Medicine, Cleveland, OH USA; 5 Geriatric Research, Education and Clinical Center, Louis Stokes Cleveland VA Medical Center, Cleveland, OH USA; 6 Infectious Diseases Section 1110W, Louis Stokes Cleveland VA Medical Center, 10701 East Boulevard, Cleveland, OH 44106 USA

**Keywords:** Bathroom, Far ultraviolet-C light, Bacteriophage MS2, *Candida Auris*, Decontamination

## Abstract

**Background:**

Aerosols generated during toilet flushing are a potential source for transmission of viral and bacterial pathogens in bathrooms. However, manual decontamination of bathrooms after each use is not feasible.

**Methods:**

We tested the efficacy of a wall-mounted far ultraviolet-C (UV-C) light technology that only delivers far UV-C when people are not present for decontamination of surfaces and aerosolized viral particles in an unoccupied hospital bathroom. A quantitative disk carrier test method was used to test efficacy against organisms on steel disk carriers placed in 9 sites in the bathroom with an exposure time of 45 min and 2 h; *Clostridioides difficile* spores were also exposed for 24 h. Efficacy against aerosolized bacteriophage MS2 was tested with a 45-minute exposure.

**Results:**

The far UV-C technology reduced methicillin-resistant *Staphylococcus aureus* (MRSA), vancomycin-resistant enterococci (VRE), *Candida auris*, and bacteriophage MS2 on steel disk carriers by *≥* 1.2 log_10_ (range, 1.2 to 4.2 log_10_) at all test sites after 2 h of exposure. The technology reduced *C. difficile* spores by < 1 log_10_ after 2 h exposure, but 4 of 9 test locations had *≥* 2 log_10_ reductions after 24 h exposure. Aerosolized bacteriophage MS2 was reduced by 4 log_10_ plaque-forming units in 45 min.

**Conclusions:**

The far UV-C light technology could potentially be useful for automated decontamination of air and surfaces in bathrooms in healthcare and community settings.

## Introduction

Contaminated bathrooms have been implicated in outbreaks of viral and bacterial infections in multiple settings, including hospitals, schools, cruise ships, airplanes, and auto dealerships [1–3]. Flushing of toilets generates large numbers of aerosol particles and droplets that may contain pathogenic organisms [1, 2, 4]. Surfaces in bathrooms can become contaminated with organisms dispersed from toilets as well as urinals, sink drains, and hands during drying, particularly if using a jet air dryer [1, 5]. Contamination can also occur through direct hand and skin or clothing contact with frequently contacted areas such as toilet seats [1]. Interventions such as closing the toilet lid when flushing or use of automatic bowl cleaners may reduce dispersal during toilet flushing [1, 2]. However, in a recent report, closing the toilet lid prior to flushing did not prevent dispersal of the non-enveloped virus bacteriophage MS2 from the toilet bowl to the toilet seat and other bathroom surfaces [6].

Manual cleaning and disinfection of bathrooms after each use is not feasible. Therefore, two recent studies have evaluated automated ultraviolet-C (UV-C) light technologies as a novel approach to address bathroom contamination. In a shared patient bathroom, a wall-mounted device that delivered a 5-minute cycle of 254-nm UV-C after each use was effective in reducing bacterial surface and aerosol contamination [7]. In a staff bathroom, a far UV-C device that turned off whenever motion was detected reduced counts of aerobic bacteria on surfaces [8]. Here, we evaluated the efficacy of a similar far UV-C technology in reducing surface contamination and aerosolized viral particles in a shared staff bathroom. Far UV-C (222-nm) was evaluated rather than 254-nm UV-C due to safety considerations. Far UV-C doses within threshold limit values proposed by the American Conference of Governmental Industrial Hygienists (ACGIH) and the International Commission on Non-Ionizing Radiation Protection (ICNIRP) may be safe in occupied areas [9–14]. Thus, accidental exposure to far UV-C, but not 254-nm UV-C, would pose minimal risk.

## Materials and methods

### Description of the far UV-C light technology

The far-UV-C technology (Mynatek, Inc., Oakland, CA) uses 3 krypton-chloride excimer lamps that emit a primary wavelength of 222 nm with a field of illumination of 60° per lamp [15]. The device includes a built-in cooling fan. The device includes proprietary sensors that detect the presence of people and/or motion in areas exposed to far UV-C. For this study, the device was programmed to automatically turn off all far UV-C delivery when people are detected in the bathroom, remain off while people are present, and resume output 30 s after they leave. In preliminary testing, it was confirmed that the device consistently turned off when personnel entered the bathroom and remained off while they were present even if they remained motionless for several minutes.

### Description of the bathroom used for testing and measurement of irradiance

**Fig. 1:**
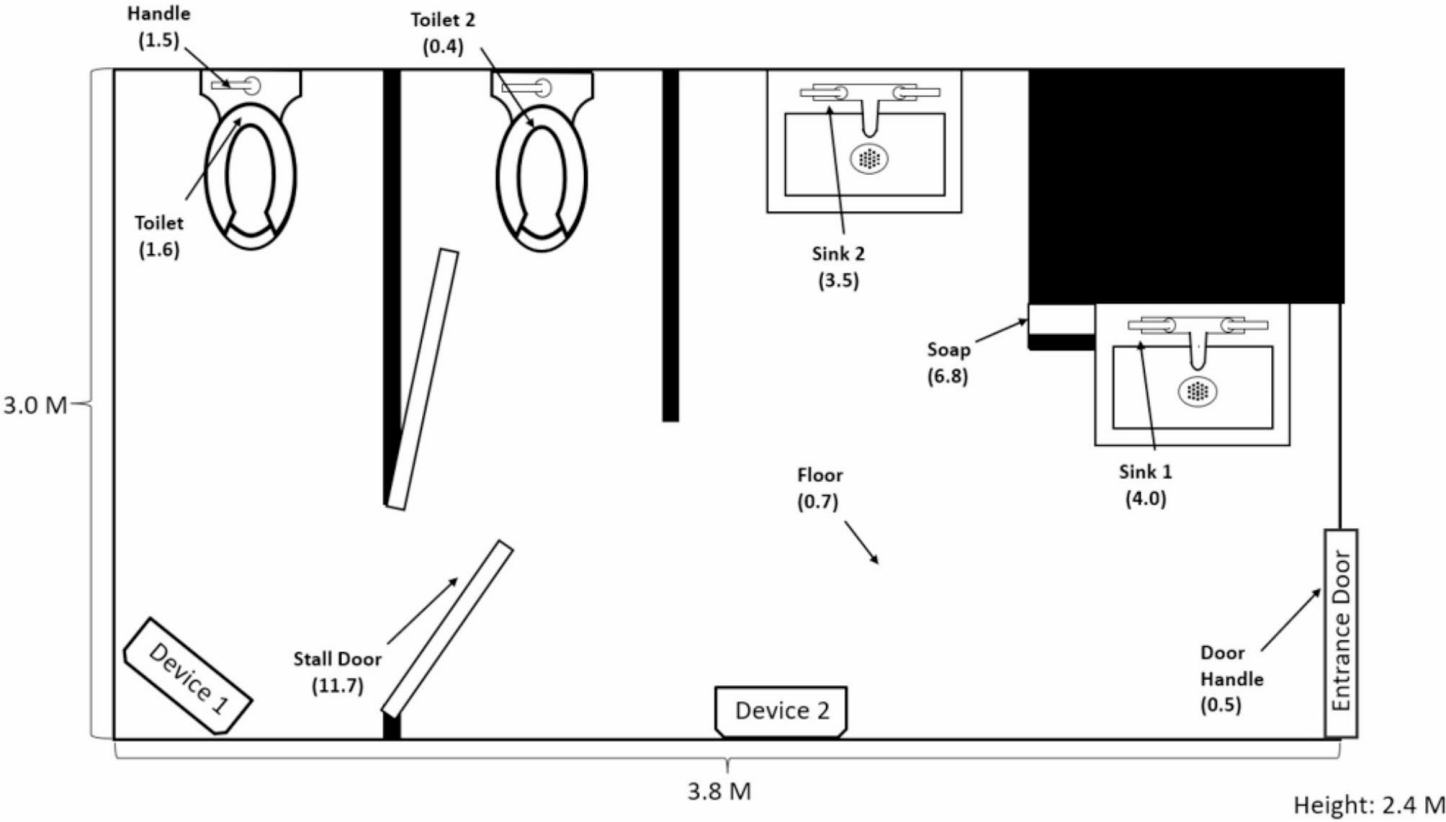
Illustration of the bathroom used for testing including the placement of the far ultraviolet-C devices and the location of nine test sites where steel disk carriers with microorganisms were placed. Irradiance readings at the test sites in µW/cm^2^ are shown in parentheses

An unoccupied staff bathroom in an unoccupied wing of the research department was used for testing. The ventilation system provides approximately 8 air changes per hour. Figure 1 provides an illustration of the bathroom including the placement of the devices and the location of nine test sites where steel disk carriers with microorganisms were placed. Two far UV-C devices were positioned just below the ceiling 2 m from the floor. The devices are intended to be mounted on the wall, but for the purposes of the study were mounted on poles. The devices were placed with the goal of minimizing shaded areas as much as was feasible with the use of 2 devices. A radiometer (UIT2400 Handheld Light Meter for 222 nm (Ushio America, Cypress, CA) was used to measure irradiance at the nine test sites with data recorded when no staff were in the room.

### Test organisms

The test organisms included a clinical isolate of pulsed-field gel electrophoresis type USA800 methicillin-resistant *Staphylococcus aureus* (MRSA), vancomycin-resistant *Enterococcus faecium* (VRE) strain C68, *Candida auris* strain AR-0385 (Clade IV; South America origin), bacteriophage MS2, and *Clostridioides difficile* American type culture collection strain 43,598. *C. difficile* spores and bacteriophage MS2 were prepared and quantitatively cultured as previously described [15, 16].

### Reduction in organisms on steel disk carriers placed in the bathroom

We tested the efficacy of the technology against the test organisms using a modification of the American Society for Testing and Materials (ASTM) standard quantitative disk carrier test method (ASTM E 2197-02) with 5% fetal calf serum as a soil load [17]. A 10 µL inoculum was spread to cover 20 mm magnetized and brushed stainless steel disk carriers. The carriers were adhered to surfaces in the test locations with 3 disks per organism at each test site. For 3 test locations (entrance door handle, bathroom stall door, and soap dispenser), the carriers were oriented vertically; for the remaining locations, the carriers were oriented horizontally. All the test locations were directly exposed to far UV-C light from one or both devices except the toilet 2 site. The disks were exposed to far-UV for 45 min and for 2 h; for *C. difficile*, the disks were also exposed for 24 h. The 45-minute continuous exposure was chosen based on previous evidence that vegetative organisms on steel disk carriers in unshaded areas were reduced by > 3 log_10_ after 45 min [15]. The 2-hour exposure was included to assess the impact of a longer exposure time on vegetative organisms; the 24-hour exposure was included for *C. difficile* because it is more resistant to killing by UV-C [15]. Control disks were prepared and processed identically and at the same time as experimental disks but were kept outside the test room to avoid far UV-C exposure. The carrier disks were processed as previously described in accordance with ASTM E 2197-02 [15–17]. All experiments were performed in triplicate. Log_10_ colony-forming unit (CFU) or plaque-forming unit (PFU) reductions were calculated in comparison to untreated controls. A reduction of *≥* 3 log_10_ in comparison to untreated controls was considered effective [16].

### Reduction in aerosolized bacteriophage MS2

We evaluated the efficacy of the far UV-C technology in reducing aerosolized bacteriophage MS2 in the bathroom as described previously [15]. A bathroom exhaust fan was operating during the experiment. For each simulation, an Aerogen Solo (Aerogen) nebulizer was used to release 1 mL of aerosol containing 10^8^ PFU of bacteriophage MS2 over 3 min. For control and test simulations, air samples were collected using NIOSH 2-stage bio-aerosol samplers (Tisch Environmental) with a flow volume of 3.5 L/min over 5-minute periods at baseline 0 to 5 min after aerosol release and 40 to 45 min after release. Control and test simulations were repeated in triplicate. Log_10_ reductions at 45 min were calculated in comparison to control experiments run in the same room with no far-UV-C exposure.

### Data analysis

Student’s *t*-test was used to compare the concentrations of bacteriophage MS2 recovered from air samples at baseline and after 45 min for control versus far UV-C simulations. For reductions in organisms on steel disk carriers, we calculated the percentage of sites achieving a *≥* 3 log_10_ reduction for each organism without performing statistical comparisons.

## Results

Irradiance readings during operation of both devices at each of the 9 test sites are shown in Fig. 1. Far UV-C light was detected at all test sites, but irradiance readings varied widely from 0.4 µW/cm^2^ on a toilet seat that was not in direct line of site of the far UV-C devices to 11.7 µW/cm^2^ on a stall door directly exposed to far UV-C and in proximity to device 1.

**Fig. 2:**
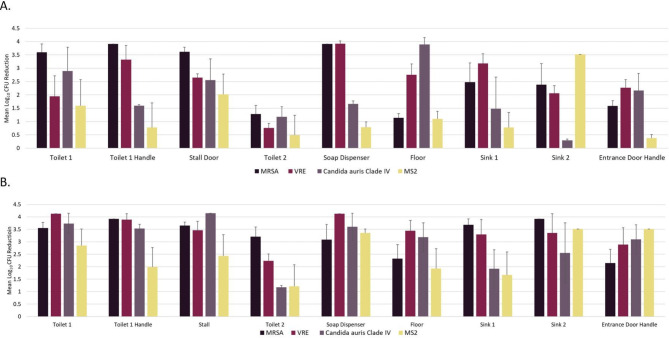
Reduction in organisms on steel disk carriers at 9 test sites in a staff bathroom after 45 min (**A**) and 2 h (**B**) of continuous exposure to far ultraviolet-C light Note. MRSA, methicillin-resistant *Staphylococcus aureus*; VRE, vancomycin-resistant *Enterococcus faecium*. Error bars show standard error. Log_10_ reductions were calculated in comparison to untreated controls

Figure 2 shows reductions of MRSA, VRE, *C. auris*, and bacteriophage MS2 on steel disk carriers after 45 min and 2 h of far UV-C exposure. After 45 min exposure, the mean log_10_ reductions varied substantially for the different test sites and organisms; mean reductions of 3 log_10_ or greater were achieved for MRSA and VRE at 4 and 3 test sites, respectively, whereas mean reductions of 3 log_10_ or greater were achieved *C. auris* and bacteriophage MS2 at only 1 site each. After 2 h exposure, *≥* 1.2 mean log_10_ reductions (range, 1.2 to 4.2 log_10_) were achieved for all organisms at all test sites; mean reductions of 3 log_10_ or greater were achieved for MRSA, VRE, *C. auris*, and bacteriophage MS2 at 7, 7, 6, and 3 sites, respectively. Notably, the toilet 2 site was not in direct line of exposure to light from either of the devices and was the site with the lowest mean log_10_ reductions for most organisms at 45 min and 2 h of exposure.

**Fig. 3:**
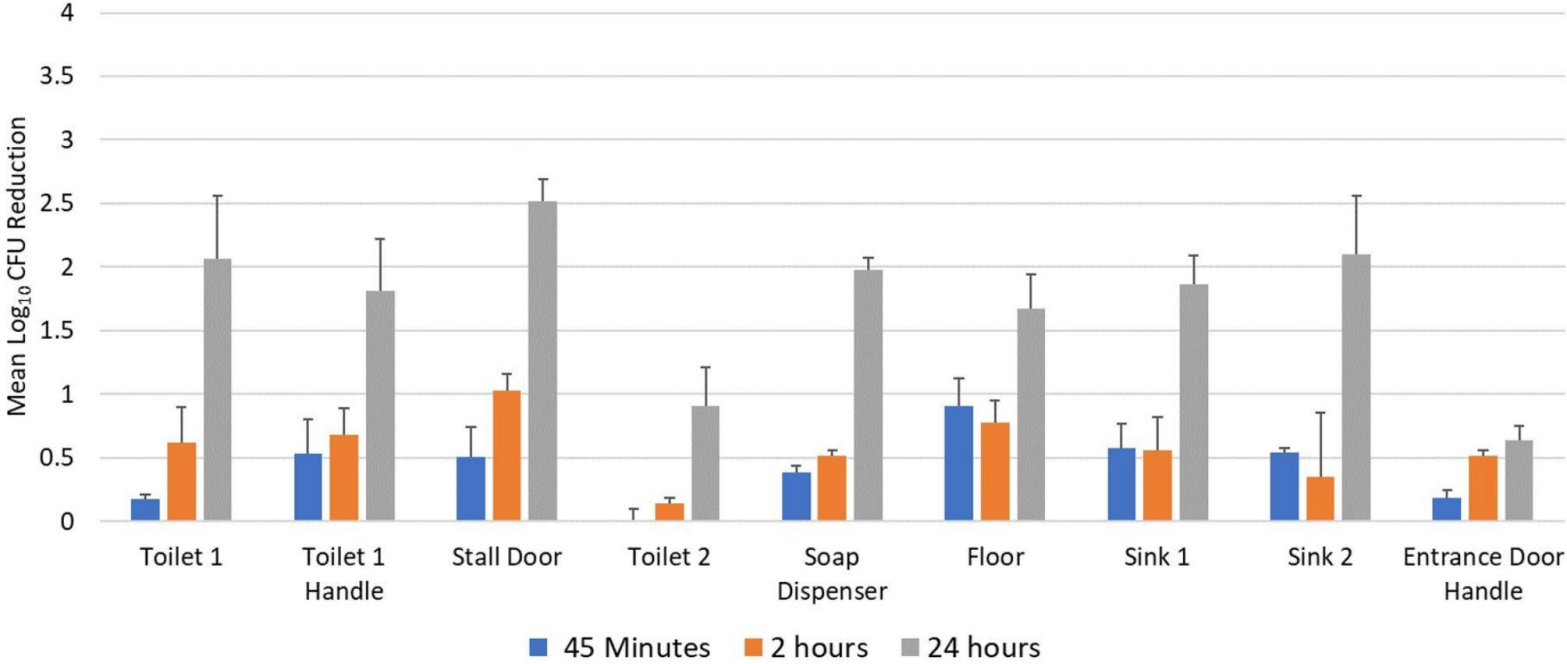
Reduction in *Clostridioides difficile* spores after 45 min, 2 h, and 24 h of far ultraviolet-C exposure in a staff bathroom. CFU, colony-forming unit

Figure 3 shows reductions in *C. difficile* spores after 45 min, 2 h, and 24 h of far UV-C exposure. After 45 min and 2 h of exposure, *≤* 1 log_10_ reductions were achieved at all sites. However, after 24 h exposure, 4 of 9 sites achieved *≥* 2 log_10_ CFU reductions; no site had a > 3 log_10_ reduction.

**Fig. 4:**
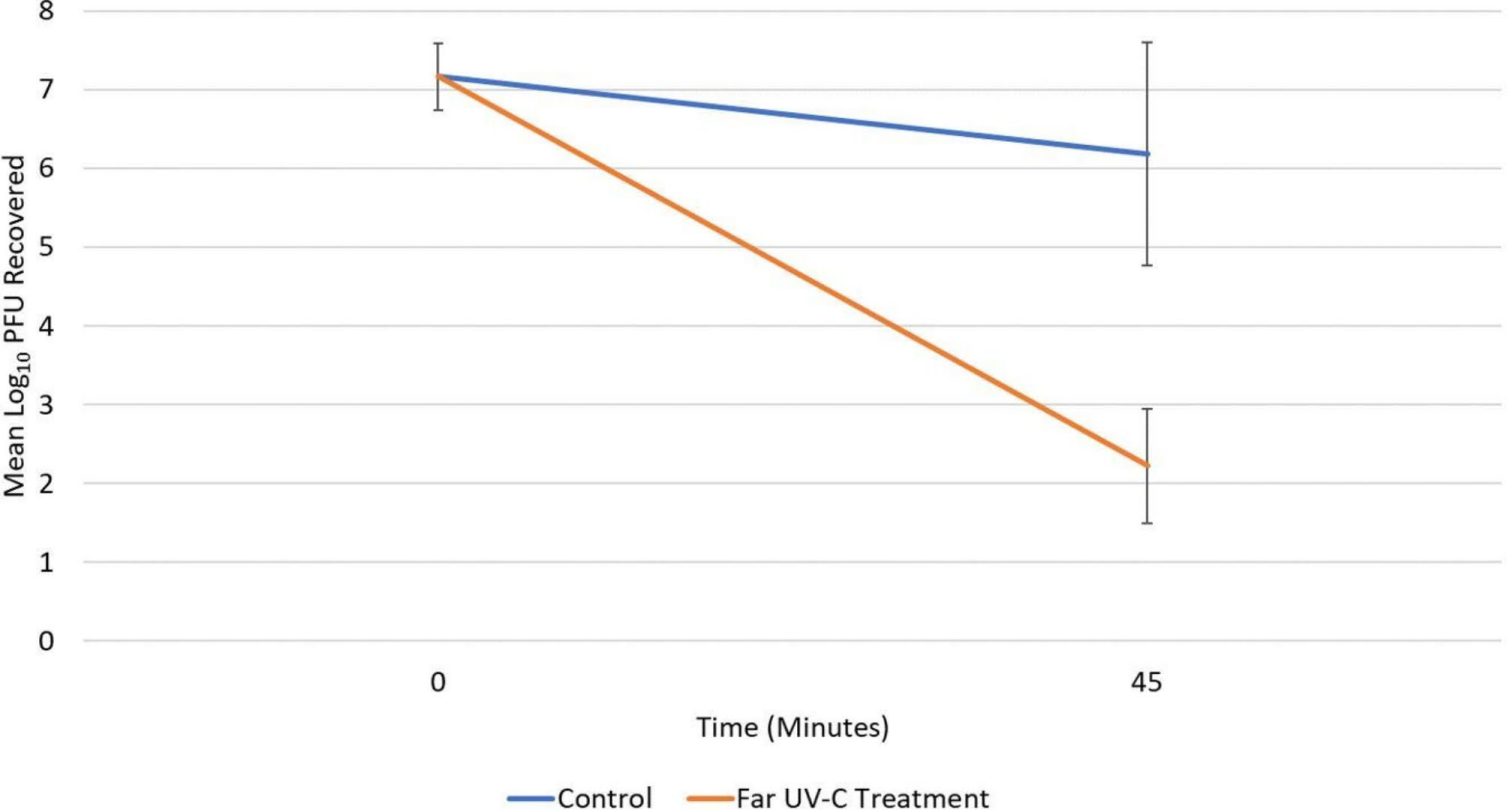
Reduction in aerosolized bacteriophage MS2 in 45 min in a staff bathroom with and without exposure to far ultraviolet-C light. Time 0 indicates values for air samples collected 0 to 5 min after aerosol release. Time 45 indicates values for air samples collected 40 to 45 min after release. Error bars show standard error. PFU, plaque-forming units

Figure 4 shows the reduction in bacteriophage MS after 45 min of far UV-C exposure. Approximately 7 log_10_ PFU of bacteriophage MS2 was recovered from air at baseline with no significant difference between control and test experiments (*P* > 0.05). After 45 min, far UV-C exposure resulted in a 4 log_10_ PFU reduction in bacteriophage MS2 in comparison to control tests with no far UV-C exposure (*P* < 0.01).

## Discussion

Technologies that provide effective, safe, and automated decontamination of air and surfaces could potentially reduce the risk for transmission of infectious pathogens in bathrooms. In the current study, we demonstrated that a technology programmed to deliver far UV-C light when people are not present reduced aerosolized bacteriophage MS2 by 4 log_10_ within 45 min in a staff bathroom. Efficacy against vegetative organisms on steel disk carriers varied substantially in different bathroom locations, consistent with reduced delivery of far UV-C light to shaded areas or sites at a distance from the light sources. However, > 3 log_10_ reductions in MRSA, VRE, and *C. auris* were achieved in most locations with a 2-hour exposure, and *≥* 1.2 log_10_ reductions (range, 1.2 to 4.2 log_10_) were achieved after 2 h for all organisms at all sites. The technology had limited efficacy against *C. difficile* spores, but with 24 h of exposure 4 of 9 test locations had *≥* 2 log_10_ reductions.

Our results suggest that the far UV-C technology could provide a useful adjunct to routine cleaning and disinfection in shared bathrooms. UV-C technologies that emit 254-nm light may achieve greater reductions with short cycle times [16]. However, the far UV-C technology has important potential safety advantages over 254-nm UV-C devices [18]. The 254-nm UV-C devices can be programmed to turn off when people enter the bathroom [7], but the potential for failure of these safety features is a concern because inadvertent exposure can be hazardous to personnel [19]. In contrast, there is a growing body of evidence that far UV-C exposure within proposed threshold limit values may be safe [9–14]. Thus, if accidental exposure to far UV-C did occur, it would likely pose minimal risk.

Several recent studies have highlighted the potential for dispersal of healthcare-associated pathogens from toilets in hospitals [4, 20, 21]. Best et al. [4] inoculated toilets with fecal suspensions containing *C. difficile* spores and demonstrated that flushing of non-covered lidless toilets resulted in dispersal to air and surfaces; closing the toilet lid reduced but did not eliminate dispersal of spores. In a pilot study conducted in rooms of patients with *C. difficile* infection, Wilson et al. [21] demonstrated increased recovery (26% versus 13%) of healthcare-associated bacteria including enterococci and *C. difficile* in post-flush versus pre-flush air samples. However, the total number of CFU recovered in individual samples was relatively low (2 to 79 CFU). Future studies are needed to examine the potential for far UV-C light technologies to reduce contamination of shared bathrooms in hospitals and nursing homes.

This study has some limitations. The experiments were conducted in an unoccupied staff bathroom. We evaluated reductions in organisms on carriers rather than on real-world surfaces. For vegetative organisms, only 45-minute and 2-hour exposures were tested. It is likely that longer overall exposure times would be achieved in real-world settings, but for bathrooms that are occupied frequently, limited far UV-C exposure might occur between individual occupants. Future studies are needed to evaluate the efficacy of the technology in real-world settings, including in patient rooms. We only included two far UV-C devices in our evaluation. Use of more than two far UV-C devices might potentially improve efficacy, particularly if this increases the proportion of high-touch surfaces in direct line of far UV-C exposure. We did not assess ozone production which is a potential concern for far UV-C technologies [9]. Thus, additional studies are needed to examined ozone concentrations in bathrooms where the far UV-C technology is used. However, there may be limited potential for ozone accumulation above recommended exposure limits in well-ventilated spaces [9]. We only tested one far UV-C technology. Additional work is needed to assess the advantages and disadvantages of far UV-C technologies that are commercially available. Finally, we did not assess the impact of far UV-C on material compatibility.

## Conclusion

Shared bathrooms have been implicated as a potential source for transmission of pathogens in healthcare and community settings. We demonstrated that a technology that delivers far UV-C light when people are not present was effective in reducing aerosolized bacteriophage MS2 and vegetative organisms in a staff bathroom. Future studies are needed to assess the efficacy of the technology in reducing contamination with pathogens in real-world settings.

## Data Availability

Datasets used and analyzed during the current study are available from the corresponding author on reasonable request.

## Abbreviations

UV-C: Ultraviolet-C
ACGIH: American Conference of Governmental Industrial Hygienists
ICNIRP: International Commission on Non-Ionizing Radiation Protection
MRSA: Methicillin-resistant *Staphylococcus aureus*
VRE: Vancomycin-resistant *Enterococcus faecium*
